# Burnout among French dermatologists: A national study

**DOI:** 10.1111/jdv.18492

**Published:** 2022-09-19

**Authors:** Nicole Jouan, Charles Taieb, Bruno Halioua

**Affiliations:** ^1^ French Federation for Continuing Education in Dermatology and Venereology (FFFCEDV) Paris France; ^2^ CHU BREST Department of Dermatology Brest France; ^3^ Patient Priority Department European Market Maintenance Assessment Fontenay‐sous‐Bois France; ^4^ Société Française des Sciences Humaines de la Peau Maison de la dermatologie Paris France; ^5^ Private Practice Department of Dermatology Paris France

Dear Editor,

The FFFCDEV (French Federation for Continuing Education in Dermatology and Venereology), which federates more than 2800 practising dermatologists (DTO), is one of the 4 official bodies of dermatology in France. Due to its missions, it gathers hospital, liberal or mixed dermatologists. A recent study evaluated the prevalence of burnout among GPs practising town practices.^1^ The FFFCDEV wanted to evaluate the prevalence of burnout among French dermatologists practising in all types of practice to study the risk and protective factors. In the framework of this evaluation, the FFFCDEV distributed a digital questionnaire including the Maslach Burnout Inventory (MBI),^2^ professional characteristics (year of thesis, exercise modalities and daily working time) and personal characteristics such as gender, marital status and number of dependents after work, sports activity or religious practice. A total of 650 dermatologists responded to the questionnaire, but only 577 fully completed the MBI and were considered evaluable. Eighty‐one per cent were women, 78.2% were self‐employed, 71.8% reported more than 35 h of practice per week, and 62.7% reported at least one encounter with aggressive patients. A total of 67.3% reported more than 20 years of practice. In total, 47.8% ([43.7–51.9] 95% CI) of French dermatologists were in a burn‐out situation: 29.3% (95% CI 25.59%, 33.01%) in mild burn‐out situation; 15.6% in moderate burn‐out situation (95% CI 12.64%, 18.56%) and 3% (95% CI 1.61%, 4.39%) in severe burn‐out situation. A total of 40.3% of male dermatologists were in a burn‐out situation vs. 49.1% of female dermatologists, and this observed difference was not statistically significant. In the absence of a secretariat, 63.2% were in a burn‐out situation versus 46% for dermatologists who had a secretariat (*p* < 0.03). There was no statistically significant difference according to the type of activity or the number of days worked. On the contrary, a difference was observed according to the number of hours worked per week (51% in a burn‐out situation vs. 40% for DTOs working more than 35 hours and less than 35 h, respectively; *p* < 0.005). Satisfaction with remuneration seems to be a catalyst for a potential burn‐out situation state: 37.8%, 47.1% and 68.6% for satisfied, moderately satisfied or not satisfied dermatologists, respectively. Finally, the fact of having been confronted with aggressive patients also seems to generate a burn‐out state. 33% of BO among those who had not been confronted, 36.5% among those who had been confronted once, and 55.5% among those who had been confronted several times. Practising a sporting activity seems to prevent the development of burnout (42.9% vs. 61.4%, *p* < 0.0001), unlike mind–body practices. (Table 1 describes the prevalence of burn‐out situations according to different indicators) We have constituted a sample representing nearly one French dermatologist out of six. A total of 47.8% were in a burn‐out situation, including 3% in a severe burn‐out situation. We are quite close to the results published for general practitioners (44.8% of French private practitioners were in a burn‐out situation, including 4.8% in a severe burn‐out situation).

**TABLE 1:** Prevalence of burnout according to different indicators

Burnout by location of practice (*p* = 0.3718)	Agglomeration of more than 50,000 inhabitants		Medium‐sized city		Rural area	
	*N*	%	*N*	%	*N*	%
Absence of burnout	161	52.3	115	50.9	20	69.0
Mild burnout	91	29.6	66	29.2	7	24.1
Moderate or severe burnout	56	18.2	45	19.9	2	6.9
Burnout by presence of a secretary (*p* = 0.0244)	Physical secretary		No physical secretary			
	*N*	*N*	*N*	%		
Absence of burnout	245	21	21	50.9		
Mild burnout	130	22	22	29.2		
Moderate or severe burnout	79	14	14	19.9		
Burnout by mode of practice (*p* = 0.2918)	Liberal activity		Mixed activity		Salaried activity	
	*N*	%	*N*	%	*N*	%
Absence of burnout	228	50,6	38	63,3	35	53,0
Mild burnout	136	30,2	16	26,7	17	25,8
Moderate or severe burnout	87	19,3	6	10,0	14	21,2
Burnout as a function of weekly working time (*p* = 0.0009)	Less than 35 h		Between 35 and 39 h		Between 40 and 45 h	
	*N*	%	*N*	%	*N*	%
Absence of burnout	67	62.6	62	63.3	105	47.1
Mild burnout	33	30.8	21	21.4	66	29.6
Moderate or severe burnout	7	6.5	15	15.3	52	23.3
Burnout by length of time on the job (*p* = 0.1046)	Under 10 years		Between 10 and 20 years		More than 20 years	
	N	%	N	%	N	%
Absence of burnout	30	52.6	79	59.9	192	49.5
Mild burnout	14	24.6	28	21.2	127	32.7
Moderate or severe burnout	13	22.8	25	18.9	69	17.8
Were confronted with demanding, aggressive or rude patients? (*p* < 0.0001)	No		Yes, once		Yes several times	
	*N*	%	*N*	%	*N*	%
Absence of burnout	72	66.7	68	63.6	161	44.5
Mild burnout	27	25.0	28	26.2	114	31.5
Moderate or severe burnout	9	8.3	11	10.3	87	24.0
Burnout as a function of income satisfaction^a^ (*p* < 0.0001)	Satisfied		Moderately satisfied		Not satisfied	
	*N*	%	*N*	%	*N*	%
Absence of burnout	145	62.2	118	52.9	38	31.4
Mild burnout	62	26.6	61	27.4	46	38.0
Moderate or severe burnout	26	11.2	44	19.7	37	30.6

^a^ The individual is considered not satisfied with his or her income when he or she gives a score of less than 3, moderately satisfied with a score between 3 and 7, and satisfied when the score given to his or her income is higher than 7.

A limited number of publications have specifically addressed burnout in dermatology and on smaller samples (between 58 and 150 responders).^3^ According to the 2019 Medscape National Physician Burnout and Depression Survey, 38% of dermatologists reported burnout,^4^ with another study reporting a prevalence of approximately 56%.^5^ The data we report with a large sample are entirely in line with published data.

The physical or verbal aggression of certain patients, which is increasingly frequent, seems to be a catalyst, along with the length of the working week and the feeling of not being sufficiently remunerated for the work carried out, whereas the practice of a sporting activity or the presence of a secretary would be protective.

## FUNDING INFORMATION

None.

## CONFLICT OF INTEREST

None.

## Data Availability

The data that support the findings of this study are available from the corresponding author upon reasonable request.
